# Human MUC4 mucin induces ultra-structural changes and tumorigenicity in pancreatic cancer cells

**DOI:** 10.1038/sj.bjc.6603868

**Published:** 2007-06-26

**Authors:** N Moniaux, P Chaturvedi, G C Varshney, J L Meza, J F Rodriguez-Sierra, J-P Aubert, S K Batra

**Affiliations:** 1 Department of Biochemistry and Molecular Biology, University of Nebraska Medical Center, Eppley Institute for Research in Cancer and Allied Diseases, University of Nebraska Medical Center, Omaha, NE 68198, USA; 2 Cell Biology and Immunology, Institute of Microbial Technology, Chandigarh, India; 3 Department of preventive and Societal Medicine, University of Nebraska Medical Center, Omaha, NE 68198, USA; 4 Department of Genetics, Cell Biology and Anatomy, University of Nebraska Medical Center, Omaha, NE 68198, USA; 5 Unité INSERM 377, Place de Verdun, 59045 Lille Cedex, France

**Keywords:** MUC4, mucin, pancreatic cancer, mitochondria

## Abstract

MUC4 is a type-1 transmembrane glycoprotein and is overexpressed in many carcinomas. It is a heterodimeric protein of 930 kDa, composed of a mucin-type subunit, MUC4*α*, and a membrane-bound growth factor-like subunit, MUC4*β*. MUC4 mRNA contains unique 5′ and 3′ coding sequences along with a large variable number of tandem repeat (VNTR) domain of 7–19 kb. A direct association of MUC4 overexpression has been established with the degree of invasiveness and poor prognosis of pancreatic cancer. To understand the precise role of MUC4 in pancreatic cancer, we engineered a MUC4 complementary DNA construct, mini-MUC4, whose deduced protein (320 kDa) is comparable with that of wild-type MUC4 (930 kDa) but represents only 10% of VNTR. Stable ectopic expression of mini-MUC4 in two human pancreatic cancer cell lines, Panc1 and MiaPaCa, showed that MUC4 minigene expression follows a biosynthesis and localisation pattern similar to the wild-type MUC4. Expression of MUC4 resulted in increased growth, motility, and invasiveness of the pancreatic cancer cells *in vitro*. Ultra-structural examination of MUC4-transfected cells showed the presence of increased number and size of mitochondria. The MUC4-expressing cells also demonstrated an enhanced tumorigenicity in an orthotopic xenograft nude mice model, further supporting a direct role of MUC4 in inducing the cancer properties. In conclusion, our results suggest that MUC4 promotes tumorigenicity and is directly involved in growth and survival of the cancer cells.

MUC4 is a high molecular weight *O*-glycoprotein produced by secretory epithelial cells for the lubrication and protection of ducts and lumen (Porchet *et al*, 1991; Khorrami *et al*, 2002). *MUC4* is expressed in various epithelial tissues including the trachea, colon, stomach, cervix, and lung (Audie *et al*, 1993, 1995). It is also expressed in goblet, ciliated, and absorptive cells in adults and in poorly differentiated cells of embryos and fetuses (Buisine *et al*, 1998, 1999). Indeed, *MUC4* is expressed early in the primitive gut, before respiratory and digestive epithelial cells have acquired their tissue and cell specificity. For these reasons, morphogenic differentiation and embryonic development functions are attributed to MUC4, and its expression can be regulated by several serum factors including retinoids (Ogata *et al*, 1992; Choudhury *et al*, 2000b), growth factors, and cytokines (Andrianifahanana *et al*, 2005).

The largest size of MUC4 allele is approximately 26.5 kb. Its deduced amino-acid sequence consists of a 27-residue peptide signal sequence followed by three imperfect repetitions of a motif varying in size from 126 to 130 residues and a unique sequence of 554 residues. The central domain is composed of a perfect repetition motif of 16 amino-acid residues, which is repeated 400 times in the largest allele. The C-terminal region contains two domains rich in N-glycosylation sites, three epidermal growth factor-like domains, a transmembrane sequence, and a short cytoplasmic tail. The MUC4 protein is hypothesised to be proteolytically cleaved at a GDPH site generating two subunits, the mucin-type subunit, MUC4*α*, and the growth factor-like subunit, MUC4*β*. MUC4 is predicted to be a membrane-associated 2.12 *μ*m long, mature protein where both subunits stay non-covalently bound.

The genomic organisation of *MUC4* is fully understood, and its characterisation has been substantially aided by the human genome project (DOE Joint Genome Institute Human Genome Project). MUC4 encompasses 25 exons, the first exon, E1, encoding the 5′-untranslated sequence, the translation start site, and the peptide signal (Escande *et al*, 2002). Exons range from 65 bp to 22 kb in size, whereas introns range from 94 bp to 16.5 kb. The largest exon, E2, is at the central position and contains a large 2.8 kb unique sequence followed by the repetition of a 48 bp motif. The tandem repeat region in E2 shows a variable number of tandem repeat (VNTR) polymorphism, and its size varies from 7.5 to 19 kb. The largest known allele for *MUC4* expresses a 26.5 kb transcript that encodes the full-length MUC4 precursor, also called sv0-*MUC4*.

MUC4 is highly expressed in human pancreatic tumours and pancreatic tumour cell lines; however, its expression is undetectable in the normal pancreas or chronic pancreatitis (Balague *et al*, 1994; Hollingsworth *et al*, 1994; Choudhury *et al*, 2000a; Andrianifahanana *et al*, 2001). MUC4 is expressed by metaplastic ducts and its expression increases with increasing grade in pancreatic intraepithelial neoplasias (Swart *et al*, 2002; Park *et al*, 2003). A recent study has shown that MUC4 is a good candidate marker for early diagnosis of pancreatic cancer in fine-needle aspirates, exhibiting a 91% sensitivity and 100% specificity (Jhala *et al*, 2006). MUC4 expression in a variety of cancer is associated with poor prognosis (Bax *et al*, 2004; Weed *et al*, 2004; Alos *et al*, 2005; Handra-Luca *et al*, 2005; Jhala *et al*, 2006). The inhibition of MUC4 expression by an antisense oligonucleotide resulted in decreased *in vivo* tumorigenicity and metastasis (Singh *et al*, 2004). Altogether, these data establish a strong association between MUC4 expression and pancreatic cancer pathogenesis. Moreover, in the rat model, sialomucin complex (SMC)/rMuc4, represses cell adhesion, blocks tumour cell killing, promotes the growth of the tumour, and is directly related to the metastatic invasion (Carraway *et al*, 2000). However, our understanding about the role of MUC4 in the development and progression of pancreatic adenocarcinoma is still unclear.

One of the hallmarks in cancer development is the inhibition of apoptosis with a central role of the mitochondria in this process. During carcinogenesis mitochondria present an enhanced aerobic glycolysis as a protective mechanism against reactive oxygen species, an acidification of the microenvironment that favours cell invasion and an enhanced resistance to mitochondria-induced apoptosis (Brand and Hermfisse, 1997; Kroemer, 2006). In addition to their role in apoptosis, mitochondria play a pivotal role in cellular metabolism, respiration, and production of ATP essential for the normal function of all human organ systems. Therefore, the mitochondrial content for any given cell is well regulated, presenting variation with the type of the cells and tissues, during cell differentiation, hormone treatment, and exercise (Robin and Wong, 1988; Renis *et al*, 1989; Shay *et al*, 1990). Increase in mitochondrial mass is directly associated with cell division and cell differentiation. In cancer cells, other roles have also been linked to increase in mitochondrial mass, including increased resistance to tamoxifen-induced apoptosis in breast cancer, further suggesting role of mitochondria in cancer progression (Dietze *et al*, 2001).

To elucidate further the functions of human MUC4, a recombinant MUC4 minigene was generated and stably transfected in pancreatic adenocarcinoma cell lines Panc1 and MiaPaCa. The mini-MUC4 was surface-localised in both the transfected cell lines. Expression of mini-MUC4 increased *in vitro* growth, motility, and invasiveness of the pancreatic cancer cells. Ultra-structural studies showed increased mitochondrial size in mini-MUC4-transfected cells, which was correlative with increased mitochondrial mass. Furthermore, overexpression of the mini-MUC4 gene in pancreatic cancer cells also resulted in an increase in tumorigenicity of these cells *in vivo.* Our results demonstrate, for the first time, a direct role of the MUC4 mucin with the cellular changes and tumour progression in human pancreatic cancer cells.

## MATERIALS AND METHODS

### Generation of mini-MUC4 gene

**Figure 1:**
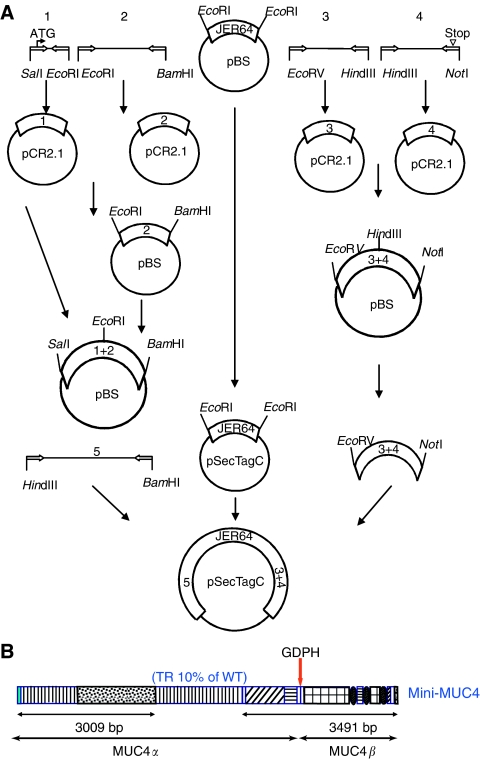
Construction of the mini-MUC4. (**A**) Schematic representation of cloning strategy for the generation of the mini-MUC4 construct. Fragments amplified by PCR were subcloned in PCR2.1 vector, sequenced, digested with specific restriction sites, and subcloned in the pBluescript vector. Finally, the 5′ region, JER64 fragment, and the 3′ fragments were subcloned in the pSecTag C vector. Each junction was sequenced to confirm the reading frame. (**B**) A schematic representation of mini-MUC4 gene. The sequence of the mini-MUC4 (320 kDa) is comparable with that of wild-type MUC4 (930 kDa), but with a repetitive domain representing only 10% the normal size.

The construction of the MUC4 minigene was performed in three steps (Figure 1A). First, the unique sequences of MUC4 were amplified by PCR: two fragments for the 5′-unique sequence, a *Sal*I/*Eco*RI product from position –71 to 178, and an *Eco*RI/*Bam*HI product from 179 to 2919 (AJ000281). Two fragments were amplified by PCR for the 3′-unique sequence, an *Eco*RV/*Hin*dIII product from position 1 to 1351 and a *Hin*dIII/*Not*I product from position 1352 to 3471 (AJ010901). Each product was cloned into pCR2.1 (Invitrogen, Carlsbad, CA, USA). Both 5′ and 3′ fragments were extracted by the introduced restriction endonuclease sites and subcloned into the pBluescript KS(+) vector (Stratagene, La Jolla, CA, USA) to get a *Sal*I/*Bam*HI 5′-full sequence and a *Eco*RV/*Not*I 3′-full sequence. In a second step, a new *Hin*dIII/*Bam*HI PCR product was performed using the 5′-full sequence as a template, and cloned into the pCR2.1 vector. This fragment is located from position 100 to 2919 (AJ00281). The *Eco*RI complementary DNA (cDNA) fragment named JER64 coding the repetitive sequence of MUC4 was extracted and subcloned into the pSecTag C vector. Finally, the *Hin*dIII/*Bam*HI 5′ fragment, as well as the *Eco*RV/*Not*I 3′-full sequence, were extracted and subcloned in frame into the pSecTag C containing the sequence repeated in tandem. At each step, PCR products were sequenced; the final construct was sequenced at different junctions to check the appropriate reading frame.

### Expression of mini-MUC4 cDNA construct

Panc1 and MiaPaCa pancreatic adenocarcinoma cell lines were transfected with plasmid DNA using the Lipofectamine method (Invitrogen). Single colonies were obtained by zeocin selection and expanded for screening. Cell lines were analysed for MUC4 protein expression by western blot with a MUC4 peptide mouse monoclonal antibody (8G7). Growth kinetic and population doubling time of transfected and control cells were determined as published previously (Singh *et al*, 2004).

### Preparation of cell lysates and immunoblotting

Cell lysates were prepared in the lysis buffer (50 mM Tris–HCl (pH 7.4), 150 mM NaCl, 1% Nonidet P-40, 0.25% Na-deoxycholate, 1 mM EDTA, 1 mM phenylmethylsulfonyl fluoride, 1*μ*g/ml of aprotinin and leupeptin, 1 mM Na_3_VO_4_, and 1 mM NaF). The protein content of the lysates was determined by using the Bio-Rad D_C_ protein assay (Bio-Rad, Hercules, CA, USA). Cell lysates were resolved on a 2% sodium dodecyl sulphate (SDS)-agarose gel, transferred to polyvinylidene difluoride (PVDF) membrane, and blocked in 5% non-fat milk in phosphate-buffered saline (PBS) for 1 h. The MUC4 antibody was diluted at 1 *μ*g/ml in PBS. The membrane was incubated for 4 h at room temperature, followed by 6 × 10 min washes in TBST (50 mM Tris–HCl (pH 7.4), 150 mM NaCl, and 0.05% Tween-20). The horseradish peroxidase (HRP)-conjugated secondary antibody was diluted at 1 : 2000 in PBS, and the membrane was incubated for 1 h at room temperature, followed by six washes in TBST. Enhanced chemiluminescence reagents were applied as per the manufacturer's instructions (Amersham Life Science, Piscataway, NJ, USA), and the blot was exposed to ECL-sensitive film (Kodak, Rochester, NY, USA).

### Confocal analysis

The cells were grown at 60–70% confluency on the sterilised coverslip. Cells were first washed with 0.1 M HEPES containing Hanks buffer and fixed in ice-cold methanol at −20°C for 2 min. Methanol-fixed cells were blocked in 10% goat serum containing 0.05% Tween-20 for 30 min at room temperature before incubation with primary antibody diluted (1 : 100) in 10% goat serum containing PBS for 60 min at room temperature. Cells were washed four times for 5 min with PBS containing 0.05% Tween-20 (PBST) and then incubated with fluorescein isothiocyanate (FITC)-conjugated goat anti-mouse secondary antibodies (Jackson Immuno Research laboratories Inc., West Grove, PA, USA) for 60 min at room temperature. Cells were again washed with PBST, five times for 5 min and mounted on a glass slide in the anti-fade VECTASHIELD mounting media (Vector Laboratories Inc., Burlingame, CA, USA). Immunostaining was observed under the Zeiss 510 LASER SCAN confocal microscope.

### Apoptosis assay

Apoptosis was measured by using the Annexin V-FITC apoptosis detection kit (Roche Diagnostics, Indianapolis, IN, USA). The cells were grown in serum-free medium and the apoptosis was detected by staining the cells with annexin V and propidium iodide solution, followed by flow cytometry.

### Cell motility assay

For motility assays, 1 × 10^6^ cells were plated in the top chamber of non-coated polyethylene teraphthalate membranes (six-well insert, pore size 8 *μ*m; Becton Dickinson, Franklin Lakes, NJ, USA). The cells were incubated for 24 h, and the cells that did not migrate through the pores in the membrane were removed by scraping the membrane with a cotton swab. Cells that transversed the membranes were stained with a Diff-Quick cell staining kit (Dade Behring Inc., Newark, DE, USA). Cells in 10 random fields of view at × 100 magnification were counted and expressed as the average number of cells per field of view. Three independent experiments were performed in each case. The data were represented as the average of the three independent experiments with the s.d. of the average indicated.

### Cell invasion assay

For Matrigel assay 1 × 10^5^ cells were seeded on Matrigel-coated membrane inserts. The bottom chamber contained 0.75 ml of Dulbecco's modified Eagle's medium (DMEM) supplemented with 10% fetal bovine serum (FBS). After incubation for 24 h at 37°C, the cells remaining inside the insert were removed with a cotton swab, and cells that penetrated the Matrigel and invaded to the lower surface of the membrane were fixed in methanol and stained using a Diff-quick reagent kit. After air-drying the membrane, the cells were visually counted at a magnification of × 100 under a microscope. Assays were performed two times in triplicate wells, and the cells present in 10 fields covering the centre of each membrane were counted. Data were expressed as the number of cells per area and as invasion index.

### Electron microscopy

Cells cultured at 70% confluence, were washed twice in ice-cold PBS, trypsinised, and fixed with 2.5% glutaraldehyde in 1 M Sorenson's phosphate buffer. The cell pellet was then subjected to overnight infiltration in Dureapam Acm. Epoxy Resin (Electron Microscopy Sciences, Fort Washington, PA, USA), flat embedding, and polymerisation at 55°C for 48 h. Embedded cells were mounted on resin stubs and sectioned at 90 nm with an Ultracut E ultramicrotome. Sections were viewed and photographed with a Zeiss 600 EM10A transmission electron microscope (TEM) at 60 kV.

### Determination of mitochondrial mass

The fluorescent dye 10-*n*-nonyl-acridine orange (NAO) (Molecular Probes, Carlsbad, CA, USA) was used to monitor the mitochondrial mass. Cells were trypsinised and resuspended in 0.5 ml of DMEM medium containing 5 *μ*M of NAO, incubated for 30 min at 37°C in the dark. After incubation, cells were kept on ice and immediately analysed by flow cytometry. A FACScan flow cytometer, equipped with a 488 nm argon laser, was used for the flow-cytometric analysis. The forward and side scatters were used to establish the size gates and to exclude cellular debris. The excitation wavelength was at 488 nm and observation at 530 nm for the green fluorescence and 585 nm for the red fluorescence.

### Tumorigenicity assay

To test the tumorigenic capacity, mini-MUC4-transfected cells, along with the control cells (parental and mock), were harvested from subconfluent cultures, and resuspended in a normal saline solution at a concentration of 4 × 10^7^ cells/ml. Single-cell suspensions of >90% viability were used for the injections. Immunodeficient mice were purchased from the Animal Production Area of the National Cancer Institute/Frederick Cancer Research and Development Center (Frederick, MD, USA). The mice were housed in specific pathogen-free conditions and were fed sterile water and food *ad libitum*. The mice were treated in accordance with the Institutional Animal Care and Use Committee guidelines. The mice (8- to 12-week old) were anaesthetised with 350 *μ*l of intraperitoneal injection of a mixture (4 : 1) of ketamine (100 mg/ml) and xylazine (20 mg/ml) diluted 10 times in sterile water. A small left abdominal flank incision was made, and the spleen was exteriorised. Tumour cells (2 × 10^6^) were injected subcapsularly in a region of the pancreas just beneath the spleen using a 1-cc U-100 insulin disposable syringe (Becton Dickinson). A successful subcapsular intrapancreatic injection of tumour cells was identified by the appearance of a fluid bleb without intraperitoneal leakage. To prevent leakage, a cotton swab was held for 30–60 s over the site of injection. Wounds were closed by making a single suture on one layer and three to five independent sutures on the outermost skin. Tumour growth was assessed by daily weighing and palpation of each animal. All mice were killed on day 60 after implantation, and the presence of metastatic lesions in different organs was determined. Pancreatic tumours were excised, weighed, and measured for their dimensions.

### Microarray analysis

RNA (60 *μ*g) from MUC4-transfected cell lines and control mock cell line were fluorescently labelled with Cy3- and Cy5-conjugated dCTP (Amersham Pharmacia, Piscataway, NJ, USA), using anchored oligo(dT) primers and Superscript II reverse transcriptase (Gibco-BRL, Gaithersburg, MD, USA). After probe generation, residual RNA was hydrolysed by treatment with NaOH and unincorporated nucleotides were removed by MicroCon YM-30 column (Amicon, Billerica, MA, USA). The latter step also serves to concentrate the labelled cDNAs. Cy3- and Cy5-labelled cDNAs were combined and diluted to 60 *μ*l with 3.5 × SSC/0.15% SDS hybridisation solution. To reduce nonspecific hybridisation, the hybridisation solution also contained 10 *μ*g of poly (dA) (Pharmacia, St Louis, MD, USA), 2.5 *μ*g of yeast transfer RNA (Sigma, Ridgefield, CT, USA), and 12.5 *μ*g of human Cot1 DNA (Boehringer-Mannheim, Ridgefield, CT, USA). The fluorescent probes were denatured and hybridised to glass slides featuring approximately 6000 cDNA elements (University of Texas Southwestern Medical Center Microarray Facility, Dallas, TX, USA) overnight at 65°C. Slides were washed successively in 1 × SSC/0.1% SDS, 1 × SSC, and 0.2 SSC for 2 min each to remove excess probe and spun dried.

Hybridised arrays were scanned for fluorescent signals using Scan Array 4000 confocal fluorescent laser scanner and accompanying ScanArray 3.0 software (Packard Instrument Company, Meriden, CT, USA). For the MUC4-transfected *vs* mock cell line plot, the log intensity in the Cy3-channel log_2_(Cy3-) and the log intensity in the Cy5-channel log_2_(Cy5-) were transformed for normalisation and visualisation purposes. A 45° counterclockwise rotation of the (log_2_Cy3, log_2_Cy5)-coordinate system and a scaling of the coordinates were applied to plot the log intensity ratio *M*=log_2_(Cy3/Cy5) *vs* the mean log intensity *A*=log_2_√RCy3Cy5. These two values were the features of interest for gene expression microarray data. A print-tip-group lowess normalisation (Yang *et al*, 2002) was applied to the plot. The lowess function is a scatter-plot smoother that performs a robust local linear fit. The printing setup of the arrayer implicated printing genes in 6 × 2 groups. Typically, systematic intensity-dependent differences between such print-tip groups are observed. Separately, for every print-tip group, a lowess normalisation was applied to the plot. The normalisation was performed by subtracting the lowess fit from the log intensity ratios M. This ensured that intensity-dependent effects were removed from the data. All data transformations were performed using the contributed R package SMA (Statistical Microarray Analysis), available from ‘Terry Speed's Microarray Data Analysis Group Page’: http://www.stat.berkeley.edu/users/terry/zarray/Html/index.html.

Calculated signal intensities and standard deviations for each feature were background-corrected, normalised to total signal intensities, and converted into ratios. The data were further analysed by imposing one or all of the following selection criteria: (1) a minimum level of expression was observed; (2) significant differential expression (twofold or greater) was observed; and (3) the expression pattern was reproducible.

### Statistical analysis

Statistical analyses were performed using SAS software (SAS Institute, Cary, NC, USA). The Mann–Whitney *U*-test was used to compare tumour weight and tumour volume between groups. Fisher's exact test was used to compare the frequency of metastasis between groups. A *P*-value <0.05 was considered to be statistically significant.

## RESULTS

### Construction of the mini-MUC4

The full-length coding sequence of *MUC4* is difficult to subclone in any known mammalian expression vector because of its dimensions and the repetitive nature of tandem repeat domain. To overcome this problem, we used the first cDNA isolated for MUC4, JER64 (Porchet *et al*, 1991), to design a mini-MUC4 construct. The JER64 fragment contains a 1.8 kb long MUC4 tandem repeat region, which is composed of the 48 bp motif in repetition. The repetitive insert was subcloned in pSecTag C vector between the *EcoR*I sites. The complete unique coding sequence and the 5′ and 3′ terminal sequences were then added in frame (Figure 1A) (detailed in the Materials and Methods section). The 8781 bp mini-MUC4 cDNA encoded an estimated 320 kDa protein, composed of 240 kDa mini-MUC4*α* subunit and the 80 kDa MUC4*β* subunit (Figure 1B). The mini-MUC4 construct harbors all the unique features of the wild-type MUC4, but contains only 10% of tandem repeat region of the main allele (Nollet *et al*, 1998). Altogether, the size of the mini-MUC4 represented 58% of the smallest and 30% of the largest MUC4 wild-type alleles, and its size was comparable to its rat homologue, SMC/rMuc4 (Carraway *et al*, 2000).

### Expression and subcellular distribution of mini-MUC4

**Figure 2:**
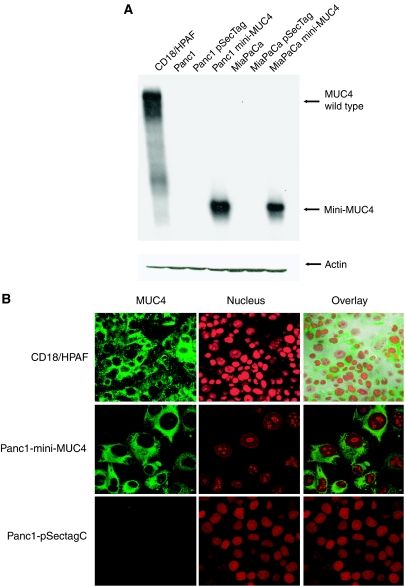
Expression and subcellular localisation of mini-MUC4 in pancreatic adenocarcinoma cell lines. (**A**) A total of 10 *μ*g protein from cell extracts were resolved by electrophoresis on a 2% SDS-agarose gel containing Tris–glycine (pH 8.8) buffer, transferred to PVDF membrane and incubated with anti-MUC4 monoclonal antibody. The membrane was then probed with HRPO-labelled goat anti-mouse Ig. Immunoblot of actin, obtained from 10% SDS-polyacrylamide gel, was used as an internal control. (**B**) Confocal photomicrograph demonstrating expression pattern of MUC4 in methanol-fixed human pancreatic tumor cells. The cells were grown at low density on coverslips for 24 h, and the acetone/methanol (1 : 1)-fixed cells were labelled using a FITC-conjugated anti-MUC4 monoclonal antibody and counterstained by propidium iodide. Magnification, × 630.

To evaluate the effects of mini-MUC4, multiple clones were selected and characterised for the mini-MUC4 expression. Mini-MUC4 was analysed by resolving the protein lysates isolated from transfected cell lines (Panc1 and MiaPaCa) on a 2% SDS-agarose horizontal gel followed by capillary transfer and immunoblotting with anti-MUC4 antibody. The immunoblotting revealed a band migrating at a distance consistent with the 320 kDa expected molecular weight of mini-MUC4 (Figure 2A). The empty vector-transfected cells were negative for MUC4 expression. CD18/HPAF, a MUC4 overexpressing cell line, was used as a positive control. The subcellular localisation of mini-MUC4 and MUC4 wild type was examined by indirect immunofluorescence and confocal laser microscopy utilising the MUC4 monoclonal antibody. CD18/HPAF cells showed both membraneous and cytoplasmic staining for MUC4 (Figure 2B). A similar distribution of MUC4 was observed for mini-MUC4-transfected Panc1 and MiaPaCa cells. Mini-MUC4 and wild-type MUC4 display a similar subcellular localisation, indicating similar processing of MUC4 and mini-MUC4 in these cells.

### MUC4 enhances the *in vitro* growth of pancreatic cancer cells

**Figure 3:**
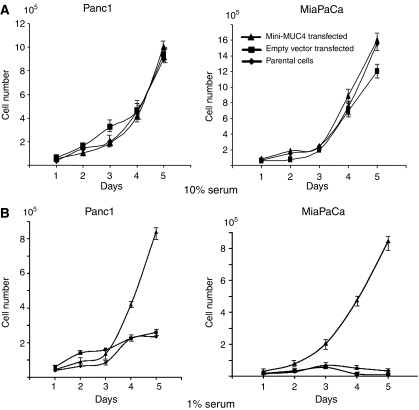
Growth kinetic patterns of the parental, vector control, and mini-MUC4-expressing cells. The cells were plated at a density of 10^4^ per well into six-well plates, grown for 6 days, and counted each day after seeding. The graph presents the result obtained in terms of the number of cells as a function of increasing duration. (**A**) The cells were maintained in medium containing 10% serum. (**B**) The cells were maintained in medium containing 1% serum.

An overexpression of MUC4 mucin has been reported in a variety of cancers. In addition, an inhibition of MUC4 mucin in pancreatic cancer cells, using an antisense strategy, diminished *in vitro* and *in vivo* growth of cancer cells. To study whether mini-MUC4 has the similar effects on growth as wild-type MUC4, a growth kinetics experiment was performed. The mini-MUC4 overexpressing cells were seeded in six-well plates in triplicate and counted for 5 days. The experiment was carried out for both Panc1 and MiaPaCa transfected with mini-MUC4, in medium containing high (10%) and low (1%) serum. No significant difference was observed for the mini-MUC4-transfected Panc1 and MiaPaca cells grown in 10% serum when compared to the control (Figure 3A). All cell lines presented similar population doubling time. However, when the cells were maintained in medium containing low serum, the mini-MUC4-transfected Panc1 cells grew much faster, with a population doubling time of 18.47 h as compared to 33 and 32.6 h for the parental and mock cells, respectively (Figure 3B). The difference was statistically significant with a *P*<0.022. The results obtained for the MiaPaCa lineage was comparable; however, we were unable to calculate the population doubling time for the parental or mock cells, the majority of the cells did not survive for more than 5 days in low serum (Figure 3B). After 5 days of growth in low serum, the difference in cell number between the control and the mini-MUC4-positive MiaPaCa cells were statistically significant with a *P*<0.013.

### MUC4 induced mitochondrial mass increase in pancreatic cancer cells

**Figure 4:**
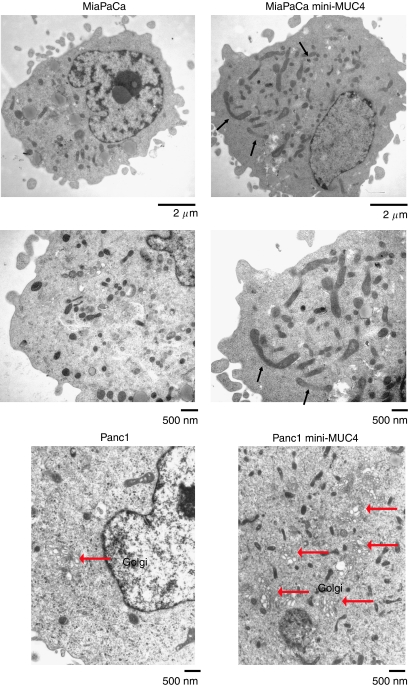
Electronmicroscopy. Cells, cultured at 70% confluence, were fixed with 2.5% glutaraldehyde in 1 M Sorenson's phosphate buffer. The cell pellet was then subjected to overnight infiltration in Dureapam Acm. Epoxy Resin (Electron Microscopy Sciences), flat embedding, and polymerisation at 55°C for 48 h. Embedded cells were mounted on resin stubs and sectioned at 90 nm with an Ultracut E ultramicrotome. Sections were viewed and photographed with a Zeiss 600 EM10A TEM at 60 Kv. The pictures presented are representative for the experiment. Twenty cells for each cell line were observed in two independent experiments. The black arrows indicate dividing mitochondria in the MiaPaCa cell line transfected with mini-MUC4 construct and the red arrows indicate the golgi complex.

**Figure 5:**
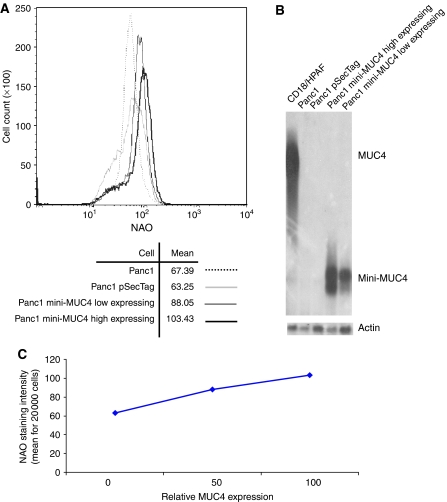
Determination of the mitochondrial mass. (**A**) The fluorescent dye NAO was used to monitor the mitochondrial mass in low MUC4-expressing (thin black curve) and high MUC4-expressing (thick black curve) Panc1 cell lines. These cell lines were compared with the parental Panc1 cells (thin grey curve) and the mock cells (discontinuous curve). The relative NAO intensity of the mini-MUC4-transfected cells (with a mean of 88.05 and 103.43 for the mini-MUC4 low and high expressing cell lines respectively) was higher than the control cell lines (with a mean of 67.3 and 63.25 for the parental and mock cell lines, respectively). (**B**) Western blot analysis of the low and high mini-MUC4 expressing cells compared with both Panc1 and Panc1 pSecTag control cell. As positive control, the HPAF/CD18 cells were used. (**C**) The NAO relative intensity was plotted depending on the mini-MUC4 expression. Mini-MUC4 expression was normalised with *β*-actin. One-hundred counts for MUC4 expression were empirically attributed to the mini-MUC4-expressing cells.

**Figure 6:**
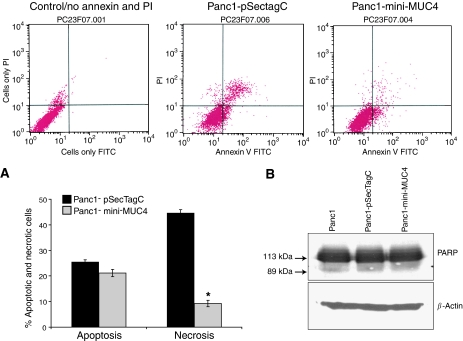
Analysis of the apoptotic and necrotic indices in mini-MUC4-overexpressing cells *vs* control cells. Panc1, either overexpressing mini-MUC4 or transfected with vector only, were serum starved 16 h to induce apoptosis. The percentage of cells undergoing apoptosis and necrosis was measured by annexin V and propidium iodide staining, respectively, followed by fluorescence-activated cell sorting analysis. (**A**) The bars represent the mean percentage of the apoptotic and necrotic cells (*n*=3, ^*^P<0.05). (**B**) Western blot analysis to show the PARP activity in Panc1, Panc1-pSecTag C, and Panc1-mini-MUC4 cell lysates. A total of 30 *μ*g protein from cell extracts were resolved by electrophoresis on a 10% SDS-polyacrylamide gel, transferred to PVDF membrane, and incubated with anti-PARP antibody. The membrane was then probed with HRP-labelled goat anti-mouse Ig. *β*-Actin was used as an internal control.

The phenotypic modifications due to mini-MUC4 overexpression were investigated by electron microscopy studies. The studies were performed for both Panc1 and MiaPaCa cells transfected with mini-MUC4 and control cells. The cells transfected for mini-MUC4 expression presented a Golgi system well developed with accumulation of numerous vacuoles, sign of active biosynthesis (Figure 4, right panel). In addition, the mini-MUC4-transfected cells showed an increased number of large, elongated, and centrally narrow mitochondria (Figure 4, right panel). The parental cells and the cells transfected with vector alone had mitochondria of normal size and shape (oval and round). The presence of elongated and centrally narrow mitochondria indicates increased mitochondrial divisions in mini-MUC4-transfected cells. The number of mitochondria examined in five fields was counted for Panc1 and Panc1 mini-MUC4 cells. Panc1 cells presented 28±14 mitochondria per field, whereas Panc1 mini-MUC4 presented 70±5 mitochondria (*P*<0.005). The mitochondrial abnormalities observed in mini-MUC4-transfected cells by electron microscopic studies were further evaluated using specific fluorescent mitochondrial probes. The fluorescent dye, NAO, which specifically binds to cardiolipin at the inner mitochondrial membrane independent of the membrane potential, was utilised to measure the changes in mitochondrial mass. To monitor the effect of mini-MUC4 overexpression on mitochondrial mass, two independent clones showing different expression levels of mini-MUC4 in Panc1 cells were investigated. The NAO uptake was compared in mini-MUC4-transfected and control cell lines. Figure 5 presents the NAO intensity measured for each cell line, the relative NAO intensity calculated for 20 000 randomly selected cells within each cell population indicated below. As shown in a table in Figure 5, the relative NAO intensity of the MUC4-transfected cells (with a mean of 88.05 and 103.43 for the cell lines expressing low and high levels of transfected mini-MUC4, respectively) was higher than the control cell lines (with a mean of 67.3 and 63.25, respectively, for the parental cells and the cells transfected with vector control). Hence, there was a progressive increase in mean NAO fluorescence of mini-MUC4-transfected Panc1 cells with increasing levels of MUC4 expression, which was also consistent with the increased number of mitochondria observed by electron microscopy. These results indicate a potential correlation between NAO uptake and MUC4 expression. The cell-cycle analysis, investigated by propidium iodide staining, showed no difference in the relative number of cells arrested at the late G_1_, G_0_/G_1_, and G_2_/M phases. The MUC4-dependent increase in mitochondrial mass appeared to be independent of the cell cycle. The difference in mitochondrial membrane potential between the mini-MUC4-transfected and control cells was analysed by utilising a lipophilic cation JC-I (5,5′,6,6′-tetrachloro 1,1′,3,3-tetraethylbenzimidazolcarbocyanine iodide dye). This mitochondrial dye exists as a monomer in solution and the monomers aggregate as a function of the mitochondrial membrane potential. The JC-I monomer fluoresces at 530±10 nm and the JC-I aggregate fluoresces at 590±17 nm. The ratio of aggregate fluorescence to monomer fluorescence is an indication of mitochondrial membrane potential. The ΔΨm was measured in mini-MUC4-expressing Panc1 cells and control cells by a ratio of 527/590 nm (data not shown). No significant difference was observed in the mitochondrial membrane potential using the JC-I dye, showing that the increase in mitochondrial mass was not associated with an increase in apoptosis. The apoptosis status of the control and mini-MUC4-corrected cells was confirmed by annexin V staining by flow cytometry and poly (ADP-ribose) polymerase (PARP) degradation by western blot (Figure 6), but no significant difference in apoptosis was observed. However, the percentage necrotic cells was significantly increased (*P*<0.05) in Panc1 cells overexpressing mini-MUC4 as compared with control cells.

### The cDNA microarray hybridisation and analysis

The variations in gene expression between the mini-MUC4-expressing Panc1 cells and mock-transfected cells were analysed by performing cDNA microarray hybridisation. A scattered pattern of expression was obtained when comparing the genes expressed in mini-MUC4-expressing Panc1 cells (presenting the highest levels of expression for mini-MUC4) *vs* the mock cell line. Among all the genes, 54 genes showed at least twofold change in expression after normalisation. The 33 genes which were overexpressed at least twofold in mini-MUC4-expressing Panc1 cells are listed in Table 1 and the 21 genes which showed at least twofold decrease in expression are listed in Table 2. Among the 33 genes overexpressed in mini-MUC4-expressing cells, four coded for proteins, which are required for mitochondrial functions. These genes are the succinyl CoA synthetase, the succinyl CoA:3-oxoacid transferase, the glycerol-3-phosphate dehydrogenase 2 (GPD2), and the isovaleryl CoA dehydrogenase (IVD). Five other genes were found to be overexpressed with a level close to the twofold cutoff: the alcohol dehydrogenase (ADH), the ATP-binding cassette transporter (ABC2), the thioredoxin reductase (TrxB2), the outer mitochondrial membrane 34 kDa translocase (TOM34), and the pyruvate kinase. Altogether, nine genes coded for proteins located and functionally active within the mitochondria. The overexpression of mitochondrial genes in mini-MUC4-transfected Panc1 was consistent with the increased number of mitochondria observed by electron microscopic studies. This indicated increased energy generating ability of MUC4-expressing cells that might enhance the tumorigenicity and metastatic potential of pancreatic adenocarcinoma cells.

### MUC4 overexpression enhances cell motility and invasiveness of pancreatic cancer cells

**Figure 7:**
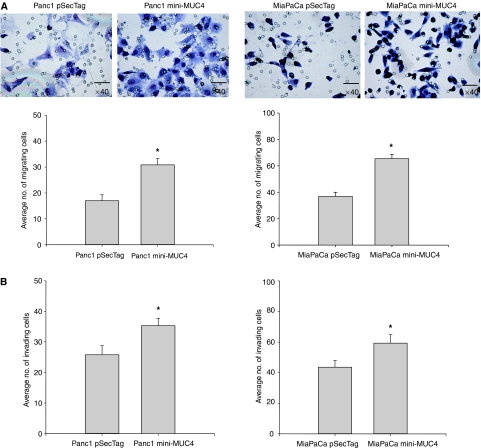
Expression of mini-MUC4 increases the motility and invasiveness of pancreatic cancer cells. (**A**) Cells were plated onto non-coated membranes for motility assays. The cells were incubated for 24 h, and those that did not migrate through the pores in the membrane were removed by scraping the membrane with a cotton swab and the remaining cells were stained. Cells that migrated through the pores were counted, and representative fields were photographed under bright field microscopy. Quantitation of cells invaded through the membrane in the above experiments is presented as a bar diagram. The mini-MUC4-expressing cells showed significant increase in cell motility as compared to control cells (^*^P<0.001). (**B**) Matrigel invasion assay to measure the invasive potential of mini-MUC4-expressing cells. The invasion of mini-MUC4-expressing and control cells through extracellular matrix was investigated using Matrigel invasion chambers. The 10% FBS was used as a chemoattractant in lower chambers. Cells migrated through the Matrigel matrix were counted, and representative fields were photographed under bright field microscopy. Quantitation of cells invaded through the Matrigel matrix in the above experiments is presented as a bar diagram. The mini-MUC4-expressing cells showed higher invasive index than the control cells (^*^P<0.05).

To examine the effect of mini-MUC4 expression on cancer cell properties, a motility and invasion assay was performed. A cell motility assay was performed by utilising uncoated porous membranes of 8.0 *μ*m pore diameter. The number of cells migrated to the lower surface of the porous membrane under chemoattractive stimulus of FBS in the lower chamber was approximately twofold greater in both mini-MUC4-expressing pancreatic cancer cells in comparison with control transfected cells (Figure 7A). An *in vitro* cell invasion assay was performed based on the principle of the Boyden chamber assay. The Matrigel matrix served as a reconstituted basement membrane *in vitro*. The cells migrating through the Matrigel matrix under chemoattractive stimulus of FBS in the lower chamber were counted and the result is presented in Figure 7B. The mini-MUC4 overexpressing cells showed significantly enhanced invasiveness (∼1.5-fold) as compared with the control cells (*P*<0.05). These data indicated that the enhanced expression of MUC4 in pancreatic cancer cells is associated with increased invasiveness.

### Overexpression of mini-MUC4 results in an increase in tumorigenicity of the Panc1 and MiaPaCa cells

To determine the effect of MUC4 expression on tumorigenicity and metastatic potential of pancreatic adenocarcinoma cells, tumorigenicity, and metastatic index were determined by transplanting the tumour cells orthotopically in the pancreas of the nude mice. We have previously reported that the inhibition of MUC4 expression by antisense technique in the well-differentiated cancer cell line, CD18/HPAF, reduced tumorigenicity, and metastasis *in vivo* (Singh *et al*, 2004). To confirm that mini-MUC4 plays a similar role in tumour development compared with the wild-type MUC4, we examined the changes in the tumorigenicity and metastatic potential of mini-MUC4-transfected Panc1 and MiaPaCa cells *in vivo.* Two million cells were implanted into the pancreas and tumour formation was checked twice a week in the first 2 weeks and daily thereafter. Primary tumours were resected, and data were recorded for tumour weight and volume (Table 3). Seventy-five percent and 64% of the mice injected with the Panc1 parental or vector-transfected control cells, respectively, developed primary tumours as compared with 100% for the cells expressing mini-MUC4 (Table 3). However, this difference in the percentage of animals harbouring tumours was not statistically significant. Among animals with tumour present, the tumour weight was significantly higher for the Panc1 cells expressing mini-MUC4 as compared with the parental cells (*P*<0.01) and the vector-transfected cells (*P*<0.01). Similar results were obtained for the tumour volume for Panc1 cells expressing mini-MUC4 *vs* Panc1 parental (*P*<0.01) and for Panc1 transfected with mini-MUC4 *vs* vector-transfected cells (*P*<0.01). No statistically significant difference was observed for the metastasis frequency. Only lymph node involvement was detected by macroscopic examination of the mice in 27% of the cases for the Panc1 cells expressing mini-MUC4 and 17–18% for the control cells. The number of macroscopic metastases per mice was comparable for all the three cell lines investigated. The results for the mini-MUC4-expressing MiaPaCa cells are not presented here, but their implantation in mice provided similar results as for the mini-MUC4-expressing Panc1 cells.

## DISCUSSION

Mucins are characterised by the presence of mucin domain, which is the largest domain and rich in serine, threonine, and proline residues. It is composed of highly glycosylated repetitive units that confer an extended conformation to the mucins due to the presence of multiple proline residues. For instance, the largest MUC4 allele identified, so far, is transcribed into RNA of 26.5 kb (Nollet *et al*, 1998). Within this 26.5 kb, 19 kb is composed of a 48 bp motif repeated up to 400 times, and this RNA encodes an estimated 2.12 *μ*m-long protein. Although the structural organisation of most of the human mucins has been elucidated, their functions in human are still not well understood. This lack of knowledge is partly due to the complexity of their transcripts and the large size of the encoded proteins. One of the classic techniques to investigate the protein functions consists of overexpression of the corresponding gene in a cell line model and to study the phenotypic changes. The large size of MUC4 RNA, however, makes this technique very difficult to perform. In this study, a mini-MUC4 construct was generated, containing the complete 5′- and 3′-unique sequences of MUC4, but with 1.8 kb of its repetitive domain instead of the original 7–19 kb. The *MUC4* minigene is 8781 bp long and encodes an estimated 320 kDa protein. It represents one-third of the estimated size of the largest *MUC4* allele and two-third of the smallest *MUC4* allele reported. Mini-MUC4 is almost equal in size to its rat homologue, SMC/Muc4 (Carraway *et al*, 2000).

In our previous studies, inhibition of MUC4 expression reduced *in vitro* growth of pancreatic cancer cells (Singh *et al*, 2004). Another study performed on rat Muc4/SMC also proves the involvement of MUC4 mucin in growth advantage to cancer cells (Komatsu *et al*, 2001). In this study, the ectopic expression of mini-MUC4 showed an increased *in vitro* growth. Our *in vivo* studies also indicated that mini-MUC4 expression is associated with increased tumorigenicity of human pancreatic cancer cells.

To evaluate the role of MUC4 in cell behavioral properties, we performed cell motility and invasion assays. The mini-MUC4 containing cells were more motile on uncoated 8 *μ*m pore membrane. A decrease in motility was observed when MUC4 was downregulated, using an antisense technology in CD18/HPAF cells, in comparison with mock-transfected cells. Furthermore, the mini-MUC4-transfected cells also presented an increased capacity to invade through the Matrigel-coated membrane. Hence, the expression of mini-MUC4 is associated with more aggressive phenotype. This property further implies that mini-MUC4 has the similar effects as wild-type MUC4 and will be valuable tool to dissect further the MUC4-mediated functions.

Our results present that MUC4 provides growth advantage to the cells and is associated with an increase in mitochondrial mass, without changing the polarity of the mitochondrial membranes. The mitochondrial mass varies with the type of the cells and tissues, and is changed during cell differentiation, hormone treatment, and exercise (Robin and Wong, 1988; Renis *et al*, 1989; Shay *et al*, 1990). An increase in mitochondrial mass is known to be related to drug treatment affecting the cell cycle (Pagliacci *et al*, 1993; Mancini *et al*, 1997). For instance, oxidative stress induced by H_2_O_2_ leads to an increase in mitochondrial mass in fibroblast. An increase in mitochondria can occur even in the absence of nuclear DNA replication (Sazer and Sherwood, 1990). Apart from that, mitochondria and mitochondrial DNA replication are also observed in the G_2_/M cell-cycle arrest. The increase in number of mitochondria occurs during cellular differentiation. The progress of differentiation in a normal cell depends on the increase in the ratio between the mitochondrial differentiation-promoting activity and decrease in nuclear-preventing activity (von Wangenheim and Peterson, 1998). During the differentiation process, the mitochondrial growth occurs at a rate that doubles its amount after each cycle. The high cytoplasmic to nuclear ratio determines the cells to differentiate, followed by apoptosis. The relationship between mitochondria and apoptosis is well established (Green and Reed, 1998). The mitochondria is known to regulate apoptosis by releasing the apoptogenic factors such as cytochrome *c* from their intermembrane space into the cytoplasm (Kluck *et al*, 1997). The release of cytochrome *c* initiates caspase activation (Liu *et al*, 1996). An increase in mitochondria mass is, therefore, considered as one of the final steps during differentiation, just prior cell death by apoptosis (von Wangenheim and Peterson, 1998). The transfection of mini-MUC4 in Panc1 cell line (a poorly differentiated cell line) increased the mitochondrial mass but was not accompanied by an increase in the apoptotic index and change in the polarisation state of the mitochondrial membrane. A similar observation has been reported in breast cancer, where resistance to tamoxifen-induced apoptosis was correlated with an increase in mitochondrial mass (Dietze *et al*, 2001). In this paper, we showed that MUC4 induces an increase in mitochondrial mass without changing the apoptotic index. However, in our MUC4 knockdown cell line model, we showed that endogenous overexpression of MUC4 is preventing apoptosis of the CD18/HPAF pancreatic cancer cells after 48–72 h serum starvation (Chaturvedi *et al*, 2007). The lack of detection of any significant change in apoptosis and PARP activity in mini-MUC4-transfected cells might be due to the fact that apoptosis was induced by serum starving the cells for less than 24 h. The shorter duration of serum starvation was applied owing to the reason that Panc1 cells and their derivatives cannot survive for more than 24 h in serum-free media. However, we found a significant upregulation in necrosis of Panc1 cells transfected with vector in comparison with mini-MUC4-transfected cells. Necrosis is a way of cell death in the absence of significant pools of cellular ATP. Despite having apoptotic signals, cells would still undergo necrosis if the ATP levels are low. Presence of mini-MUC4 by itself might be providing survival signals and preventing the apoptosis; however, due to lack of survival signals in the absence of mini-MUC4 and due to reduced levels of ATP in the vector containing cells, these cells might undergo necrosis (Bernardi *et al*, 1999; Denecker *et al*, 2001). The mechanism and the pathways involved in this phenomenon still need to be determined. Thus, results suggest that MUC4, by increasing mitochondrial mass, most probably contributes to the redox status of the cells, which in turn provides the cancer cells with a survival advantage in pre-angiogenesis, leading to increased tumour progression. This advantage was observed when the cells were grown in medium containing low serum concentration. Control cells were dying over time, whereas the MUC4-expressing cells survived and expanded.

Microarray analysis performed using the mini-MUC4 cellular model revealed overexpression of numerous genes coding for mitochondrial proteins in mini-MUC4-expressing cells (Table 1). These upregulated genes code for various enzymes that regulate different mitochondrial functions, such as citric acid cycle, ketoacidosis, glycolysis, amino acid, and lipid metabolism. None of the deregulated genes code for proteins implicated in other mitochondrial functions, such as apoptosis. Therefore, it appears that the mini-MUC4-mediated increase in mitochondrial mass is directly associated with an increase in metabolic activity. Mitochondrial mass regulation is a key point of numerous physiologic processes, including embryonic development, fat metabolism, and ageing. Our results present MUC4 as a partner in the regulation of these vital physiological steps. MUC4, at the surface of the epithelial cells, might act as an active sensor for a differentiation pathway. Some of the genes downregulated in mini-MUC4-overexpressing cells were annexin I, apolipoprotein, collagen type XIV, CD53, CD9 antigen, metastasis associated (*mta 1)* and vascular cell adhesion molecule 1 (Table 2). The *mta 1* and vascular cell adhesion molecule are known to be involved in the metastasis processes (Langley *et al*, 2001; Hofer *et al*, 2004). The nonsignificant changes in metastasis may be explained in part by downregulation of these genes in mini-MUC4-transfected cells.

One of the hypotheses that would need to be further investigated is the resistance to hypoxia-induced apoptosis driven by the increase in mitochondrial mass after mini-MUC4 overexpression. Cancer cells overexpressing MUC4 might use this strategy to survive and establish themselves before angiogenesis takes place. Some evidence is provided by the fact that only tumorigenicity was increased by mini-MUC4 overexpression and not the metastatic index. This observation suggests that an increase in tumorigenicity is associated to the unique sequence of MUC4 (MUC4*β* for instance) and the increase in metastatic index is related to the size of the large repetitive domain present in MUC4*α*.

In conclusion, our data provide evidence supporting the role of MUC4 mucin in the progression of pancreatic cancer. We demonstrate that MUC4 enhances the growth, motility, and invasive properties of pancreatic cancer cells *in vitro*. Overexpression of MUC4 mucin also induces cellular changes and tumour progression of pancreatic cancer cells. Hence, MUC4 can be a useful target in the development of novel therapeutic strategies for the treatment of pancreatic cancer.

## Figures and Tables

**Table 1:** Genes overexpressed in the mini-MUC4-transfected cells in comparison with the control cells

**Accession number**	**Name**	**Fold upregulated**
W38923	Transmembrane receptor (ror2)	4.08
N90368	Succinyl-CoA synthetase	4.01
R25074	Transmembrane 4 superfamily protein (SAS)	3.61
W60015	Translation initiation factor eIF-2*α*	3.57
N72452	RAD52 (*Sachharomyces cerevisiae*) homolog	3.54
H11692	Neuron-specific vesicle coat protein and cerebellar degeneration antigen (*β*-NAP)	3.50
T51895	Hepatoma transmembrane kinase	3.44
R59579	Prostaglandin D2 synthase	3.30
AA490680	Transcobalamin II	3.19
H07991	Vascular endothelial growth factor related protein VRP	2.99
H29256	Neuronal DHP-sensitive, voltage-dependent calcium channel *α*-1D subunit	2.97
AA410680	Tropomodulin	2.88
AA453105	Histone 2A-like protein (H2A/l)	2.81
AA037014	Prostaglandin transporter hPGT	2.75
H11792	Putative splice factor transformer2-*β*	2.70
R22274	Phosphoethanolamine cytidylyltransferase	2.69
N80617	Multidrug resistance-associated protein homolog (MRP5)	2.45
H54686	Mitogen-responsive phosphoprotein (DOC-2)	2.38
AA491302	Islet cell autoantigen 1 (69 kDa)	2.30
T64134	Monocyte chemoattractant protein-4 precursor (MCP-4)	2.26
AA457051	Lymphoid-restricted membrane protein (Jaw1)	2.26
R40897	Succinyl CoA:3-oxoacid CoA transferase precursor (OXCT)	2.19
AA489743	Interferon-inducible 56-kDa protein l	2.16
AA482128	Protein-tyrosine kinase tyk2 (non-receptor)	2.10
AA169176	Glycerol-3-phosphate dehydrogenase 2 (mitochondrial)	2.09
N79051	Transmembrane tyrosine-specific protein kinase ROS1	2.08
AA464149	Isovaleryl coenzyme A dehydrogenase	2.08
AA489714	*Homosapiens* mRNA for ZYG homologue	2.08
H37761	Mitogen induced nuclear orphan receptor (MINOR)	2.06
AA455712	Kruppel-related zinc finger protein (ZNF184)	2.03
W23757	Keratin 13	2.01
AA521453	Human flightless-I	2.01
R19878	Reelin (RELN)	2.00

**Table 2:** Genes downregulated in the mini-MUC4-transfected cells in comparison with the control cells

**Accession number**	**Name**	**Fold upregulated**
H63077	Annexin I (lipocortin I)	5.33
AA478589	Apolipoprotein E	4.64
AA167222	Collagen type XIV	2.91
H60549	Ribosomal protein S26	2.89
T94626	Fibrinogen *γ*-A chain precursor	2.74
H77766	Metallothionein	2.72
R43544	Ribosomal protein L32	2.59
H71847	Flavin-containing monooxygenase 2	2.58
R09561	Decay accelerating factor for complement	2.52
AA489569	Mesothelial keratin K7 (type II)	2.51
AA284668	Urokinase-type plasminogen activator	2.50
AA132090	CD53 antigen	2.43
AA412053	CD9 antigen	2.36
H72722	Metallothionein I-B gene	2.26
T96083	Low-Mr GTP-binding protein (RAB31)	2.26
R05336	*β*-1,4 *N*-acetylgalactosaminyltransferase	2.21
T72877	Interleukin-1 receptor antagonist	2.14
AA598478	Complement component 7	2.13
N71159	Metastasis-associated mta1	2.11
R94153	Inositol 1,4,5-trisphosphate 3-kinase B	2.03
H16637	Vascular cell adhesion molecule 1	1.84

**Table 3:** Growth of pancreatic tumours developed by orthotopic implantation of Panc1 control (parental and vector-transfected) and mini-MUC4-expressing cells in immunodeficient mice

**Cell line**	**Mice with tumour (%)**	**Median tumour weight (mg)**	**Median tumour volume (mm^3^)**	**Mice with metastases (%)**
Panc1 mini-MUC4	11/11 (100%)	1050±150^a^	770±55^a^	3/11 (27%)
Panc1	9/12 (75%)	235±35	28±5	2/12 (17%)
Panc1 pSecTag C	7/11 (64%)	145±20	20±2	2/11 (18%)

Mice were injected orthotopically with 2 × 10^6^ cells from Panc1, vector transfected and mini-MUC4-expressing cell lines in beige nude mice.

Mice were killed at day 60 post-injection and tumours were resected, weighed, and their dimensions were measured.

The mini-MUC4-expressing cells showed a significant increase in tumour weight, tumour volume but no significant difference in metastasis was observed.

a *P*<0.0001 compared with Panc1 pSecTag C.
